# Can artificial intelligence help for scientific writing?

**DOI:** 10.1186/s13054-023-04380-2

**Published:** 2023-02-25

**Authors:** Michele Salvagno, Fabio Silvio Taccone, Alberto Giovanni Gerli

**Affiliations:** 1 Department of Intensive Care, Erasme Hospital, Université Libre de Bruxelles, 1070 Brussels, Belgium; 2 Department of Clinical Sciences and Community Health, Università Degli Studi di Milano, 20122 Milan, Italy

**Keywords:** Artificial intelligence, Scientific writing, Machine learning, Chatbots

## Abstract

This paper discusses the use of Artificial Intelligence Chatbot in scientific writing. ChatGPT is a type of chatbot, developed by OpenAI, that uses the Generative Pre-trained Transformer (GPT) language model to understand and respond to natural language inputs. AI chatbot and ChatGPT in particular appear to be useful tools in scientific writing, assisting researchers and scientists in organizing material, generating an initial draft and/or in proofreading. There is no publication in the field of critical care medicine prepared using this approach; however, this will be a possibility in the next future. ChatGPT work should not be used as a replacement for human judgment and the output should always be reviewed by experts before being used in any critical decision-making or application. Moreover, several ethical issues arise about using these tools, such as the risk of plagiarism and inaccuracies, as well as a potential imbalance in its accessibility between high- and low-income countries, if the software becomes paying. For this reason, a consensus on how to regulate the use of chatbots in scientific writing will soon be required.

## Introduction

A chatbot is an electronic system (generally a software) that simulates conversations by responding to keywords or phrases it recognizes and that can be integrated into various platforms, such as websites, mobile apps, and messaging platforms.

The Chatbot Generative Pre-trained Transformer (ChatGPT), developed by OpenAI, is a type of Artificial Intelligence (AI) software designed to simulate conversations with human users. This chatbot works through algorithms programmed to understand natural language inputs and answer with appropriate responses, either pre-written or newly generated by the AI. ChatGPT is constantly improved with reinforcement techniques, natural language processing, and machine learning, to improve its ability to understand and thoroughly respond to users’ needs. Concretely, you can conversationally ask anything and receive a fast and adequate human-like written reply to your questions or requests such as: (a) write a small text on a given topic; (b) get information on a topic of interest; (c) compose an email or message with a certain tone, specific content, and intended for a particular person; (d) correct the shape of a text or change its wording; (e) solve problems.

As such, this chatbot could also be used in scientific writing [1]. Indeed, ChatGPT could become a promising and powerful tool for tasks such as automated draft generation, article summarizing, and language translation, which may be useful in academic activities to make writing work faster and easier. However, the use of this tool in scientific writing raises some ethical concerns and should therefore be regulated.

## ChatGPT in scientific writing

ChatGPT is already able to help medical researchers and scientists to write articles and abstracts, in literature research, to summarize data or information, to provide suggestions for structure, references, and titles, in language reviews to make the text more readable, or even to generate a full draft of a paper [2]. At this stage, although feasible, no article dealing with critical care medicine has been published so far using this application. Moreover, whether writing a manuscript dealing with a topic of critical care medicine would be easier or more difficult when compared to other medical disciplines remains unknown. Which are the limits of possible applications of these tools have to be clarified.

ChatGPT can generate text on a wide variety of topics. Recently, an entire letter to the editor was written by ChatGPT, answering the author's questions [1]. Artificial Intelligence cannot generate new ideas, but it can organize and develop the ones of the researcher, creating an initial draft. However, this appears to be a starting point for a human-based development of the text, as the automatic text generated is far from being a substitute for the knowledge, creativity, and critical thinking of human experts.

**Fig. 1:**
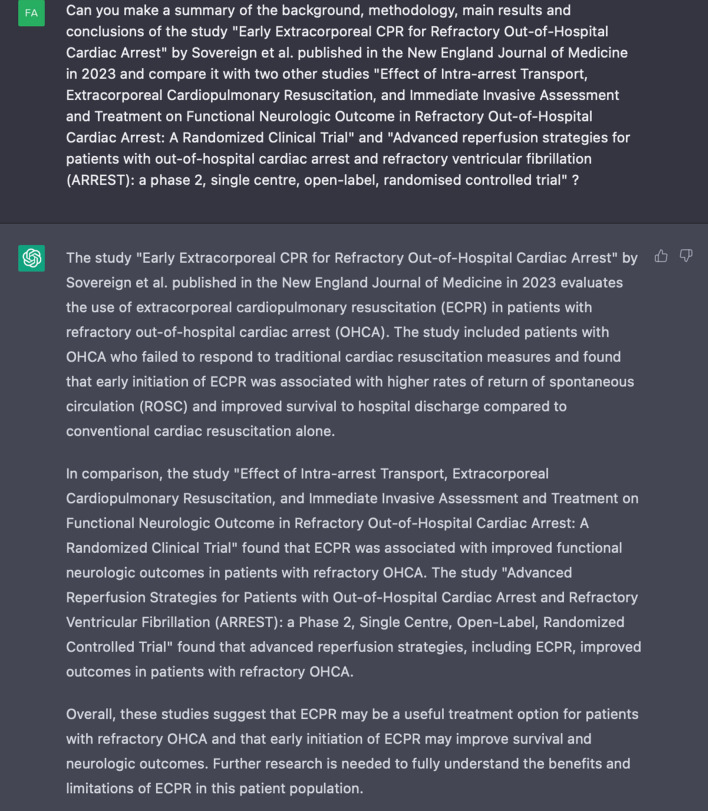
Example of a summary of the existing literature on a specific topic

For the literature search, ChatGPT and AI research assistants (such as “elicit.org”) can aid researchers in the review process by finding academic papers, summarizing their conclusions, and highlighting areas of uncertainty (i.e., summarizing the recent randomized clinical trial on the use of extracorporeal membrane oxygenation for refractory cardiac arrest [3], Fig. 1). This could help physicians, as an example, to quickly gain an understanding of the current state of knowledge on a particular topic, and to identify potential gaps that need to be addressed, although the provided summary might be quite generic and do not critically analyze differences among studies.

During the writing process, ChatGPT can help to generate an initial draft of a scientific paper and even suggest titles. By providing raw information, ChatGPT can also assist in composing the section on the methods used in the study, justify sample size and describe data analysis techniques. From author’s experience, when the manuscript has been finalized, ChatGPT is extremely effective for the editing process; formatting and language editing, rewriting a particularly complex sentence in a clearer way, and even summarizing the entire text in order to compose a suitable abstract, are feasible using this approach, although the results are not always satisfactory, but they certainly save time. ChatGPT has been recently asked to prepare regular discharge summaries [4], but the quality of the content has not been specifically evaluated. To our knowledge, a structured evaluation to determine the quality of the output has not been performed yet. More complicated writing processes, such as systematic review and meta-analyses, require the human intervention and ChatGPT might be helpful mainly for editing.

A future potential application of AI is in the automatic generation of figures, tables, and other visual elements of the manuscript, which can aid in summarizing data. These elements are important for the clarity and understanding of the manuscript, but they are often time-consuming to create.

Importantly, the writing process of a scientific paper requires, for the moment, the guidance and supervision of human researchers who are experts in the field to ensure the accuracy, coherence, and credibility of the content before being used or submitted for publication. Chatbots can help but needs the researcher’s input, and inadequate inputs would lead to inadequate results. For this reason, chatbots and AI, in general, should not replace human researchers' expertise, judgment, personality, and—in the end—responsibility.

## Chatbots vs. human beings

As AI, ChatGPT has a superior advantage in terms of fast comprehending information deeply and connecting evidence to reach conclusions, compared to humans who have limitations in their ability to read a comprehensive range of literature and distinguish the connections between seemingly separate pieces of information.

Moreover, it may be difficult to recognize whether a paper is written by a chatbot or a human being [5], because chatbots use advanced techniques, such as natural language processing (NLP) and machine learning, to generate text that is similar to human writing. To detect the author is a complex task and requires thorough critical reading to reach a conclusion. However, a few characteristics might reveal that a paper was written by a chatbot, such as the lack of nuance, style, or originality, which could allow the identification by AI output detectors and skeptical human reviewers [6]. Interestingly, the same writing traits could be found in texts written in a language that is not an individual's native tongue. By focusing on these traits, it is possible that AI plagiarism detectors would identify non-native English language papers as AI-generated text. It would be interesting to find these tools' sensibility in detecting the authors of texts written by these two groups.

Nevertheless, the chatbot-generated text might lack the subtle phrasing and word choice that a human author might use to convey a certain meaning or tone. It might also be vaguer and contain inconsistencies that would not be present in a human-written paper. Instead, if the paper contains a high degree of structural and grammatical errors, it may suggest that it was written by a human being (but it should not be the other way around). Lastly, if the paper is discussing a topic that is very specific and highly technical, it is less likely that a chatbot could generate such a text, as it would require a deep understanding of the topic and the ability to generate scientific analysis and conclusions.

## Ethical considerations

Ethical concerns could also limit the use of these chatbots for scientific writing [7].

The process of acquiring knowledge from others and writing new or review articles involves humans incorporating both what they have learned from others and their ideas. It is natural for humans to repeat the findings, statements, and written works of others, and thus to come close to committing plagiarism by presenting an idea without proper referencing to the original authors. AI or ChatGPT systems can commit plagiarism under this definition but can also be programmed to avoid copying others by rephrasing their work in a manner similar to what human authors do. However, using programs to reformulate sentences and writing to reduce the percentage of plagiarism (i.e., asking the software to rewrite a section written by other authors with different words) could not be considered acceptable in scientific research. If we define “plagiarism” as a mere act to copy someone else work, just rephrasing what it was written, regardless of the method used, and without adding anything personal, it is a violation of academic integrity. For this reason, journal editors should use programs to detect written content using AI to detect plagiarism better.

Second, the lack of an expert and critical human mind behind scientific work (which is the basis of the scientific method) could lead to a risk of perpetuating or amplifying existing biases and inaccuracies in the data, providing unfair results and hampering scientific growth. Whatever the use of AI, we believe that the presence of an expert in the field in conducting scientific activity and writing is a necessary cornerstone even to guarantee the quality of the work.

Third, the incredible development of AI tools can lead to a significant increase in publication numbers from some researchers, but not accompanied by a real increase in her or his experience in that field. Ethical issues can therefore arise regarding hiring professionals by academic institutions that score on the number of publications rather than on their quality.

Fourth, whether the ChatGPT should be mentioned within the authors of the manuscript written using this approach remains not adequately defined. Finally, if at the moment ChatGPT and other chatbot services are free of charge, it is not guaranteed that they will not become paying in the future. The introduction of charges to access these chatbots could lead to a further disparity between high- and low-income countries (as well as between more junior to older professionals), for scientific production, resulting in unfair facilitation for the formers with unpredictable consequences.

## Chatbot as a tool in the ICU

In addition to scientific writing, ChatGPT has the potential to assist physicians in their hospital work by saving time and allowing them to focus on providing patient care. In an Intensive Care Unit (ICU), where constant monitoring of multiple patient information is required, such as treatment progression, laboratory values, microbiological results, and fluid balance calculation, ChatGPT can assist in several ways.

First, ChatGPT can provide general information about recognized ICU protocols: when given a specific request, ChatGPT would be able to generate a response (i.e., initial management of sepsis) by analyzing the input and identifying patterns in the text data that it has been trained on. Furthermore, ChatGPT has a distinct advantage over humans in quickly gathering, comprehending, and utilizing information. In future, it may eventually be taught to apply this knowledge in clinical practice by presenting the latest evidence to healthcare professionals, if not directly creating patient-tailored protocols to reflect new findings.

Currently, it could already be used for generating clinical notes by just providing raw information, such as daily summaries and discharge summaries, which could save time and increase accuracy. Moreover, it could help in communicating with patients and families, translating into other languages, and generating personalized messages with accurate and timely information about the patient's condition, which would not have the purpose of replacing, but add to, the doctor-patient (or doctor-relatives) relationship. Unfortunately, depending on the applicable legislation, privacy issues may arise when providing patients' information.

Anecdotally, when provided with clinical information, laboratory values, past medical history, and other relevant data, ChatGPT may already assist physicians in, for example, determining appropriate treatment options; however, it is important to note that the answers provided by the chatbot can be erroneous or dated, as the technology is still in development and may not have the level of accuracy, current knowledge, or precision that is desired. Nevertheless, it is believable that AI will continue to improve in the future and potentially provide more accurate and reliable results.

As next future perspectives, AI in general could potentially be trained to automatically extract and understand all the relevant information from electronic health records and by analyzing patient data, such as vital signs, laboratory results, and medical history, helping doctors to quickly access patient information, providing recommendations for interventions, and allowing more fast and comprehensive decisions. The implementations of these AI systems, which already exist in several areas of the world, with chatbots, could create an interacting system which would provide immediately elaborated information to the treating physician.

## Conclusions

ChatGPT is an AI software potentially able to assist in the writing process of a scientific paper and can help in the literature review, identify research questions, provide an overview of the current state of the field, and assist with tasks, such as formatting and language review. Moreover, it can have applications in clinical practice as time-savers. As chatbot tools will be widely adopted in the near future, it is crucial to have international academic regulations in place to regulate their use in scientific writing and establish mechanisms for identifying and penalizing unethical usage. Chatbots are simply tools, they can assist human researchers but should not be used as a replacement for human researchers' expertise, judgment, and personality.

We have requested to ChatGPT “Could you review this paper and tell us if you (as an author!) agree to submit it to Critical Care Journal?”. The (non-modified) response from ChatGPT has been: “*As an AI model, I am not able to review or submit papers to journals as I am not a researcher or author. However, I can give you some feedback on the paper you've provided. The paper presents a clear and comprehensive overview of the potential uses of ChatGPT in scientific writing, specifically in the field of critical care medicine. The introduction provides a good background on chatbots and ChatGPT and how it can be used in various tasks. The paper goes on to discuss the potential uses of ChatGPT in scientific writing, such as draft generation, literature research and language review. The paper also touches on the ethical concerns and limitations of using ChatGPT in scientific writing. I would recommend that you review the paper again, check for grammar and spelling errors, and make sure that the citations and references are correctly formatted. It would be also important to have a proper conclusion that summarizes the main ideas of the paper and the future perspectives. It's important to keep in mind that ChatGPT should always be used in combination with the expertise and judgement of human experts and its output should be validated before it is used in clinical practice.”*

## Data Availability

Not applicable.
