# Correction to: Can artificial intelligence help for scientific writing?

**DOI:** 10.1186/s13054-023-04390-0

**Published:** 2023-03-08

**Authors:** Michele Salvagno, Fabio Silvio Taccone, Alberto Giovanni Gerli

**Affiliations:** 1 Department of Intensive Care, Erasme Hospital, Université Libre de Bruxelles, 1070 Brussels, Belgium; 2 Department of Clinical Sciences and Community Health, Università Degli Studi di Milano, 20122 Milan, Italy

**Correction to** **:** **Critical Care (2023) 27:75**

**https://doi.org/10.1186/s13054-023-04380-2**

Shortly after initial publication of this article [1], the authorship and Acknowledgements were updated in line with Springer Nature authorship policies. Large Language Models (LLM), such as ChatGPT, do not currently satisfy our authorship criteria. An attribution of authorship carries with it accountability for the work, which cannot be effectively applied to LLMs [2].

The correct author byline reads:

Michele Salvagno, Fabio Silvio Taccone & Alberto Giovanni Gerli

The revised Acknowledgements read:

For the writing of this article, we have not received funds or support from OpenAI, which is not associated with the entire process that led to the preparation of this article. The text, written with the support of the ChatGPT by OpenAI, has however been modified by the human authors, who assume full responsibility for form and content.

The authorship list, Acknowledgements and Author contributions have been updated in the original article.

## References

[1] Salvagno, M., ChatGPT, Taccone, F.S. et al. Can artificial intelligence help for scientific writing?. *Crit Care* **27**, 75 (2023). 10.1186/s13054-023-04380-2 10.1186/s13054-023-04380-2 PMC9960412 36841840

[2] *Nature* **613**, 612 (2023) 10.1038/d41586-023-00191-1

